# Histiocytic cell neoplasms involving the bone marrow: summary of the workshop cases submitted to the 18th Meeting of the European Association for Haematopathology (EAHP) organized by the European Bone Marrow Working Group, Basel 2016

**DOI:** 10.1007/s00277-018-3436-0

**Published:** 2018-08-06

**Authors:** Alexandar Tzankov, Markus Kremer, Roos Leguit, Attilio Orazi, Jon van der Walt, Umberto Gianelli, Konnie M. Hebeda

**Affiliations:** 1 Institute of Pathology, University of Basel, Hospital, Schönbeinstrasse 40, 4055 Basel, Switzerland; 2 Pathology, Städtisches Klinikum München, Sanatoriumsplatz 2, 81545 Munich, Germany; 3 Department of Pathology, University Medical Center Utrecht, H04-312, POB 85500, 3508 GA Utrecht, Netherlands; 4 Division of Hematopathology, Department of Pathology and Laboratory Medicine, Weill Medical College of Cornell University, 525 E, 68th Street, New York, NY 10021 USA; 5 Department of Histopathology, Guy’s and St Thomas’ Hospitals, Westminster Bridge Road, London, SE1 7EH UK; 6 Pathology Unit, Department of Pathophysiology and Transplantation, University of Milan and Fondazione IRCCS, Ca’ Granda - Maggiore Policlinico, Via Francesco Sforza 35, 20122 Milan, Italy; 7 Department of Pathology 824, Radboud University Medical Center, POB 9101, 6500 HB Nijmegen, The Netherlands

**Keywords:** Bone marrow biopsy, Erdheim-Chester disease, Histiocytic sarcoma, Mutation, Myeloid neoplasm, EAHP workshop

## Abstract

The bone marrow is a preferential site for both reactive and neoplastic histiocytic proliferations. The differential diagnosis ranges from reactive histiocyte hyperplasia in systemic infections, vaccinations, storage diseases, post myeloablative therapy, due to increased cell turnover, and in hemophagocytic lymphohistiocytosis, through extranodal Rosai-Dorfman disease to neoplasms derived from histiocytes, including histiocytic sarcomas (HS), Langerhans cell histiocytoses (LCH), Erdheim-Chester disease (ECD), and disseminated juvenile xanthogranuloma (JXG). One of the most important recent developments in understanding the biology of histiocytic neoplasms and in contributing to diagnosis was the detection of recurrent mutations of genes of the Ras/Raf/MEK/ERK signaling pathway, in particular the *BRAF*^V600E^ mutation, in LCH and ECD. Here, we summarize clinical and pathological findings of 17 histiocytic neoplasms that were presented during the bone marrow symposium and workshop of the 18th European Association for Haematopathology (EAHP) meeting held in Basel, Switzerland, in 2016. A substantial proportion of these histiocytic neoplasms was combined with clonally related lymphoid (*n* = 2) or myeloid diseases (*n* = 5, all ECD). Based on the latter observation, we suggest excluding co-existent myeloid neoplasms at initial staging of elderly ECD patients. The recurrent nature of Ras/Raf/MEK/ERK signaling pathway mutations in histiocytic neoplasms was confirmed in 6 of the 17 workshop cases, illustrating their diagnostic significance and suggesting apotential target for tailored treatments.

## Introduction to histiocytic neoplasms involving the bone marrow

The bone marrow (BM) symposium of the 18th meeting of the European Association for Haematopathology (EAHP) in Basel in September 2016 (EAHP 2016) was dedicated to non-lymphoid/non-myeloid cell proliferations in the BM, including histiocytes, mast cells, and dendritic cells; the last were summarized elsewhere [1]. In the current paper, we summarize the cases of histiocytic neoplasms involving the BM which were submitted to the workshop.

**Fig. 1:**
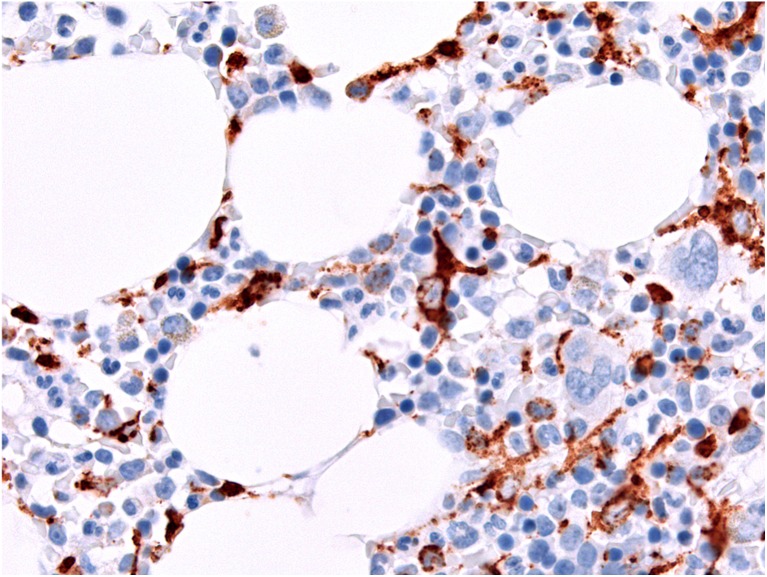
Histological appearance and distribution of histiocytic cells in a normal bone marrow highlighted by a CD68 stain, × 360. Note a “stellate” cell with dendroid protrusions surrounded by erythroid precursors in the center. Since CD68 stains lysosomes, the staining appears dotted

The BM, as a source of histiocytic precursor cells and a major home to tissue histiocytes and macrophages, which constitute approximately 10% of its microenvironment, is commonly affected by reactive and neoplastic histiocytic disorders [2], the latter occurring either primarily or as secondary spread. Histiocytes are usually dispersed throughout the BM (Fig. 1), located particularly in the erythropoietic islands as nurse-like cells. They are inconspicuous unless they are increased in number, enlarged, show a disturbed location (clustering, bone apposition), or because of altered cytoplasm due to phagocytosis [3]. Non-neoplastic diffuse histiocyte accumulations in the BM are associated with increased cellular turnover (sea-blue/pseudo-Gaucher cells, e.g., in myeloproliferative neoplasms, idiopathic thrombocytopenic purpura, thalassemia), systemic infections, *bacillus Calmette-Guérin* vaccination, myeloablative therapy (foamy cells), storage disorders, accompanying plasma cell neoplasms (crystal storing histiocytosis), and T cell proliferations in hemophagocytic lymphohistiocytosis (HLH). Several reactive examples were submitted to the EAHP workshop but are not addressed in this report, which focuses on BM involvement by neoplasms of histiocytic origin.

Histiocytoses, i.e., neoplasms derived from histiocytes, are rare disorders characterized by the accumulation of macrophages, dendritic cells, or monocyte-derived cells in various tissues and organs. Except for clear-cut malignant proliferations composed of atypical cells showing an infiltrative/destructing growth pattern, tumor necrosis, anisocytosis, anisokaryosis, increased nucleo-cytoplasmic ratio, chromatin abnormalities, hypereosinophilic nucleoli, and brisk or atypical mitotic activity, the distinction of histiocytoses from reactive histiocytic proliferations such as those listed above might be challenging, since some histiocytoses may display only minimal morphological deviation. The importance of the integration of clinical (e.g., a syndromic context) and radiologic features (e.g., osteolysis, “hairy kidney”; see later) in the diagnosis cannot be overemphasized. The diagnostic value of the aberrant expression of various mutant or phosphorylated proteins, such as V600E mutant *BRAF* [4], phosphorylated extracellular signal-regulated kinases (*ERK1*/2) [5], or, as recently suggested, the enzymatic subunit of the polycomb repressive complex 2 (*EZH2*) [6], still requires validation, but seems promising.

One of the most important developments contributing to the understanding of the biology of histiocytic neoplasms was the detection of recurrent mutations of genes of the Ras/Raf/MEK/ERK signaling pathway, in particular, the *BRAF*^V600E^ mutation in Langerhans cell histiocytosis (LCH) and Erdheim-Chester disease (ECD) [7, 8]. Mutually exclusive with *BRAF*^V600E^, additional genetic aberrations in the same pathway include mutations of *MAP2K1* and *ARAF* in LCH [9, 10], and *KRAS*, *NRAS*, and *PIK3CA* and—very rarely—*ARAF* in ECD [11, 12]. Recently, recurrent oncogenic mutations of components of the MAPK pathway, including *KRAS*, *SMAD4*, and *MAP2K1* (but not *BRAF*), have been reported in a few cases of, particularly extranodal, Rosai-Dorfman disease (RDD) [11, 13, 14], challenging its assumed non-neoplastic nature in extranodal presentation. Interestingly, individuals suffering from the autoimmune lymphoproliferative syndrome, lymphoproliferative disorders associated with germline *FAS* and *RAS* mutations, can also show RDD-like features [15, 16], and new concepts (see later) of RDD suggest that nodal and extranodal forms may represent different disorders, which may explain the discrepant evidence for both a non-clonal and clonal nature of RDD lesions.

A novel classification of histiocytoses has been recently proposed [12], defining five subgroups based on clinical and/or phenotypical criteria, namely (1) Langerhans-type histiocytoses; (2) cutaneous and mucocutaneous histiocytoses; (3) malignant histiocytoses; (4) the group of HLH/macrophage activation syndromes, which is defined by a combination of symptoms, morphological findings and laboratory tests [17]; and finally (5) RDD. This classification differs slightly from the current World Health Organization (WHO) classification of histiocytic and dendritic neoplasms, which is primarily based on morphological and phenotypical criteria [18] and recognizes (1) histiocytic sarcomas, (2) tumors derived from Langerhans cells (LC), (3) indeterminate dendritic cell tumors, (4) interdigitating and (5) follicular dendritic cell sarcomas, (6) fibroblastic reticular cell tumors, (7) disseminated juvenile xanthogranulomas (JXG), and (8) ECD, while RDD has not been included in this classification.

*RDD* is a heterogeneous entity, and classic cases (single or regional lymph node involvement with a self-limiting clinical course) and extranodal cases probably represent distinct disorders. For now, it is recommended to make a clear distinction between these two forms [12]. Accumulation of large histiocytic cells with hypochromic, roundish nuclei with often prominent nucleoli and pale cytoplasm with abundant emperipolesis is highly characteristic of RDD. RDD histiocytes express S100, CD4, CD11c, CD14, CD68, and CD163, while CD1a and/or langerin is negative. Involved tissues usually contain abundant polyclonal plasma cells, particularly IgG4-positive types, B cells, and increased fibers. RDD can also be observed at several extranodal sites including the bones/BM [19, 20]. In our personal observations, RDD of the bone accounts for approximately 0.3% of all symptomatic bone lesions [Tzankov unpublished]. A RDD case with aggressive morphological features, involvement of multiple lymph nodes and diffuse skeletal spread was submitted to the workshop (Table 1, case 15).

**Table 1:** Case summary

ID Submitter	Sex/age (years)	Disease spread	Diagnosis / panel diagnosis	Clinical presentation	Positive markers	Negative markers	Genetics	Follow-up
“True” histiocytic sarcomas								
1 Dr. Jiang	M/28	Liver, skin, lymph nodes, testes	Histiocytic sarcoma / AML with monocytic differentiation	B symptoms Back pain	CD4, CD31, CD33, CD56, CD68, CD163	CD30, CD34, CD117, CD123, MPOX, TCL1, TdT, tryptase	Not performed	Dead 8 mo after HD-CTx + allo-SCTx due to allo-SCTx-related complications
2 Dr. Lee	F/51	Liver, spleen	Histiocytic sarcoma	B symptoms Hemophagocytosis	CD31, CD68, CD163, fascin, lysozyme	CD1a, CD34, S100, MPOX	46,XX	n.a.
3 Dr. Geyer	M/77	Liver, spleen, lungs, gastro-intestinal tract, lymph nodes	Histiocytic sarcoma	Dyspnea, fatigue Eosinophilia Hemophagocytosis	CD68, CD163, HLA-DR	CD1a, CD21, langerin, lysozyme, S100, tryptase	46,XX No mutations by means of a 21 gene myeloid panel	Autopsy case
Histiocytic sarcomas and clonal histiocytic disorders accompanying/arising in other neoplasms								
4 Dr. Kuzmanov	M/32	Spleen, skin, bone marrow	Histiocytic sarcoma (HS) with concurrent mediastinal mixed germ cell tumor (MGCT)	B symptoms Hemophagocytosis MGCT	CD4, CD68, S100	CD1a, CD56	i(12p) in both the HS and MGCT	n.a.
5 Dr. Fernandez-Pol	F/77	Liver, bone marrow	Clonally related histiocytic sarcoma arising in a patient with low grade follicular lymphoma (FL)	FL in the BM	CD4, CD43, CD56, lysozyme	CD34, CD68, CD117, CD163, MPOX, TdT	*BCL2* rearrangement in FL and HS	n.a.
6 Dr. Alobeid	M/2	Bone marrow Subsequent spread to the skin, liver, spleen, and pancreas	Atypical histiocytic proliferation with juvenile xanthogranuloma (JXG) phenotype after remission of T-ALL	Worsening thrombopenia after complete remission of T-ALL Subsequent spread to the skin, liver, spleen and pancreas	CD14, CD68, CD163, FXIIIa, fascin	BRAF^V600E^, CD1a, S100, langerin	T-ALL: TCR clonal and del(9)(p13) JXG lesion: polyclonal, no del(9)(p13)	Dead 9 mo after T-ALL diagnosis and 26 d after JXG-like lesion diagnosis due to allo-SCTx-related complications
Erdheim-Chester (and related) diseases								
7 Dr. Lee	M/54	Multiple bones, orbital fat, retroperitoneum	Erdheim-Chester disease	Bilateral proptosis	α-1 antitrypsin, CD68, fascin	CD1a, S100, lysozyme	Not performed	Steroids, methotrexate, chemotherapy, INF-α Alive > 10 years
8 Dr. Hamza	M/37	Multiple bones	Erdheim-Chester disease	Knee pain Generalized joint pain	CD68	CD1a, CD45, S100	No cytogenetics No *BRAF*^V600E^, *NRAS*, and *PIC3CA*	None
9 Dr. Reichard	F/63	Multiple bones	Erdheim-Chester disease	Hip pain Generalized bone pain B symptoms	CD68	CD1a, S100	46,XX *BRAF* ^V600E^	None
10 Dr. Wong	M/70	Lungs, mesenterium, retroperitoneum	Erdheim-Chester disease with concurrent clonally related myeloid neoplasm / Hemophagocytic lymphohistiocytosis (HLH) associated with undefined myeloid neoplasm	Dyspnea, fatigue B symptoms Hemophagocytosis Disseminated intravascular coagulation	BRAF^V600E^, S100	CD1a, langerin	No cytogenetics PB and lesions: *ASXL1, BRAF*^V600E^, *TET2*, *U2AF1*	Dead within 3 d
11 Dr. Zhou	M/67	Multiple bones, pericardium, pleura, retroperitoneum, mesenterium	Erdheim-Chester disease with concurrent clonally related CMML	Pleural and pericardial effusion Neutrophilia and monocytosis	CD68, CD163, FXIIIa	S100	46,XY PB and lesions: *BRAF*^V600E^, *SRSF2*, *TET2*	Stable disease on prednisone
12 Drs. Hoehn and Ozkaya	M/66	Multiple bones, soft tissues, kidney, CNS	Erdheim-Chester evolving to clonally related CMML	Generalized bone pain B symptoms	CD68, CD163	ECD: CD34, CD117, S100	46,XY No *BRAF*^V600E^ *NRAS* ^Q61R^ in both ECD and CMML	Steroids, INF-α 2 years later decitabine for CMML 1 year later *MEK *inhibitor considered
13 Dr. Aqil	M/80	Lungs, retroperitoneum	Erdheim-Chester disease evolving to clonally related AML	Dyspnea on exertion 2 years later leukocytosis and thrombopenia	ECD: BRAF^V600E^, CD68, FXIIIa, S100 AML: BRAF^V600E^, CD4, CD33, CD68, CD163, lysozyme, MPOX	ECD: CD1a AML: CD1a, CD34, CD61, CD117, FXIIIa, S100	No cytogenetics *BRAF* ^V600E^	Steroids 2 years later chemotherapy for AML
14 Dr. Roth	M/44	Unknown	Erdheim-Chester disease (no histology provided) with subsequent AML	Pancytopenia Documented history of ECD	ECD: CD14, CD163, FXIIIa, fascin AML: CD13, CD33, CD34, CD117, HLA-DR, MPOX, TdT	ECD: S100 AML: CD14, CD36, CD56, CD64	AML: 46,XY No *BRAF*^V600E^, *FLT3*-ITD, *FLT3*^D835^, *NPM1*	IFNα for 3 years until development of AML
Other malignant histiocytic disorders involving the bone marrow								
15 Dr. King	F/49	Multiple lymph nodes, diffuse skeletal involvement	Rosai-Dorman disease with aggressive features	Generalized bone pain B symptoms Visual disturbance	CD163, S100	CD1a	46,XX	Prednisone -> cladribine with stable disease for 1 year
16 Dr. Grogg	M/74	Innumerable bone lesions	Mixed Langerhans cell (LHC)/non-LHC sarcoma / Langerhans cell sarcoma with aberrant expression of CD163	Back pain Anemia with leukoerythroblastosis Pathologic bone fractures (equivocally due to concurrent plasma cell neoplasm)	LHC component: BRAF^V600E^, CD1a, langerin, S100 Histiocytic component: BRAF^V600E^, CD163		46,XY *BRAF* ^V600E^	Dead of disease after 3 mo
17 Dr. Wu	F/0.5	Spleen, skin	Non-Langerhans cell histiocytosis with HLH / HLH associated with disseminated JXG	Hemophagocytosis Splenomegaly	CD163 Skin: CD68, CD163	BRAF^V600E^, CD1a, CD21, CD56, CD123, langerin, S100	46,XX, add(2)(q35), add(5)(q35), add(15)(q15) No *BRAF*^V600E^, *NF1*, myeloid- (48 gene panel) or HLH-associated mutations	HLH protocol treatment with resolution and cytogenetic remission

Abbreviations: *AML*, acute myeloid leukemia; *allo-SCTx*, alogeneic stem cell transplantation; *CMML*, chronic myelomonocytic leukemia; *d*, days; *F*, female; *HD-CTx*, high-dose chemotherapy; *INF-α*, interferon-α; *mo*, months; *M*, male; *n.a.*, not available; *T-ALL*, T cell acute lymphoblastic leukemia; *TCR*, T cell receptor

*JXG*, especially the solitary dermal JXG, is probably the most common histiocytosis [12, 21] and represents a benign proliferative disorder of children. It is typically characterized by the presence of “Touton” giant cells and a distinct, but non-specific immunophenotype (FXIIIa+/CD1a-/CD14+/CD68+/S100±). Mutations of MAPK pathway genes in JXG are increasingly reported [22–24]. There is an association with neurofibromatosis type 1, especially for the disseminated form [25]. Disseminated JXG is much rarer than the solitary form. It can affect the BM, and an interesting case of an atypical histiocytic proliferation with JGX phenotype after remission of T cell lymphoblastic leukemia (T-ALL) (Table 1, case 6) and one of HLH associated with disseminated JXG (Table 1, case 17) were submitted to the workshop.

*LCH* is a proliferation of LC with characteristic morphology (oval cells with abundant slightly eosinophilic cytoplasm with central grooved/indented nuclei with fine chromatin), ultrastructure (Birbeck granules), and phenotype (CD1a+/CD68+/langerin+/S100+), accompanied by eosinophils, lymphocytes, and macrophages, as well as osteoclast-type giant cells. Clinical presentation varies from an asymptomatic solitary lesion to a lethal multisystem disease [26]. LCH typically involves bones and the skull bones are the most frequently affected. The debate on the nature of LCH is now resolved by the discovery of the recurrent mutually exclusive *BRAF*^V600E^ [7] or *MAP2K1* mutations in over 80% of cases [9]. As already mentioned, this was a milestone towards understanding the pathobiology of histiocytic neoplasms in general. Several recent findings suggest that LCH has a heterogeneous histogenesis [27]. Multisystemic cases may be neoplastic disorders derived from aberrant hematopoietic progenitor cells that have acquired a *BRAF*^V600E^ mutation, which may explain the multifocal bone/BM involvement [28]. Indeed, in seven patients suffering from LCH, the *BRAF*^V600E^ mutation was also detected in their CD34+ hematopoietic progenitor cell pool [29]. One patient with metachronous LCH and Hodgkin lymphoma bearing the *BRAF*^V600E^ mutation has been reported [30] and there is evidence from a case submitted to the workshop (Table 1, case 16) as a mixed LC/non-LC sarcoma—as suggested by the submitter—that has been classified by the panel as LC sarcoma with aberrant expression of CD163. These cases illustrate the plasticity of LCH histogenesis.

On the other hand, skin-limited LCH in children is probably derived from tissue-restricted LC precursors, in keeping with the benign behavior of this variant [31]. Patients with low risk and unifocal LCH lack mutated progenitor cells in the peripheral blood, pointing to heterogeneity in the cell of origin of this disease and the prognostic importance of the particular affected cellular pool involved by the respective driver mutation [29, 31].

*HS* has a particularly pronounced association with neoplasms derived from other non-histiocytic lineages, since a substantial number of cases is associated with other hematolymphoid tumors [especially follicular lymphomas (FL); Table 1, case 5] [32–35] and HS may display B or T cell clonality [35, 36]. HS can also arise in mediastinal germ cell tumors [37, 38] as illustrated by case 4. The distinction of HS with BM involvement from acute myeloid leukemia (AML) with monocytic differentiation is difficult and depends on integration of morphology (large, usually pleomorphic cells > 20 μm in diameter with abundant cytoplasm [18]) and phenotypic characteristics, particularly the absence of the myeloid markers CD33 and myeloperoxidase and positivity for at least one histiocytic marker (i.e., either CD4, CD11c, CD14, CD68, CD163, or lysozyme), considered as a prerequisite for the diagnosis of HS, as shown in case 1. Druggable mutations of genes of the Ras/Raf/MEK/ERK signaling pathway are recurrent in HS in general [39, 40], as illustrated by several cases submitted to the 2016 EAHP workshop on histiocytic neoplasms involving the lymph nodes [35].

**Fig. 2:**
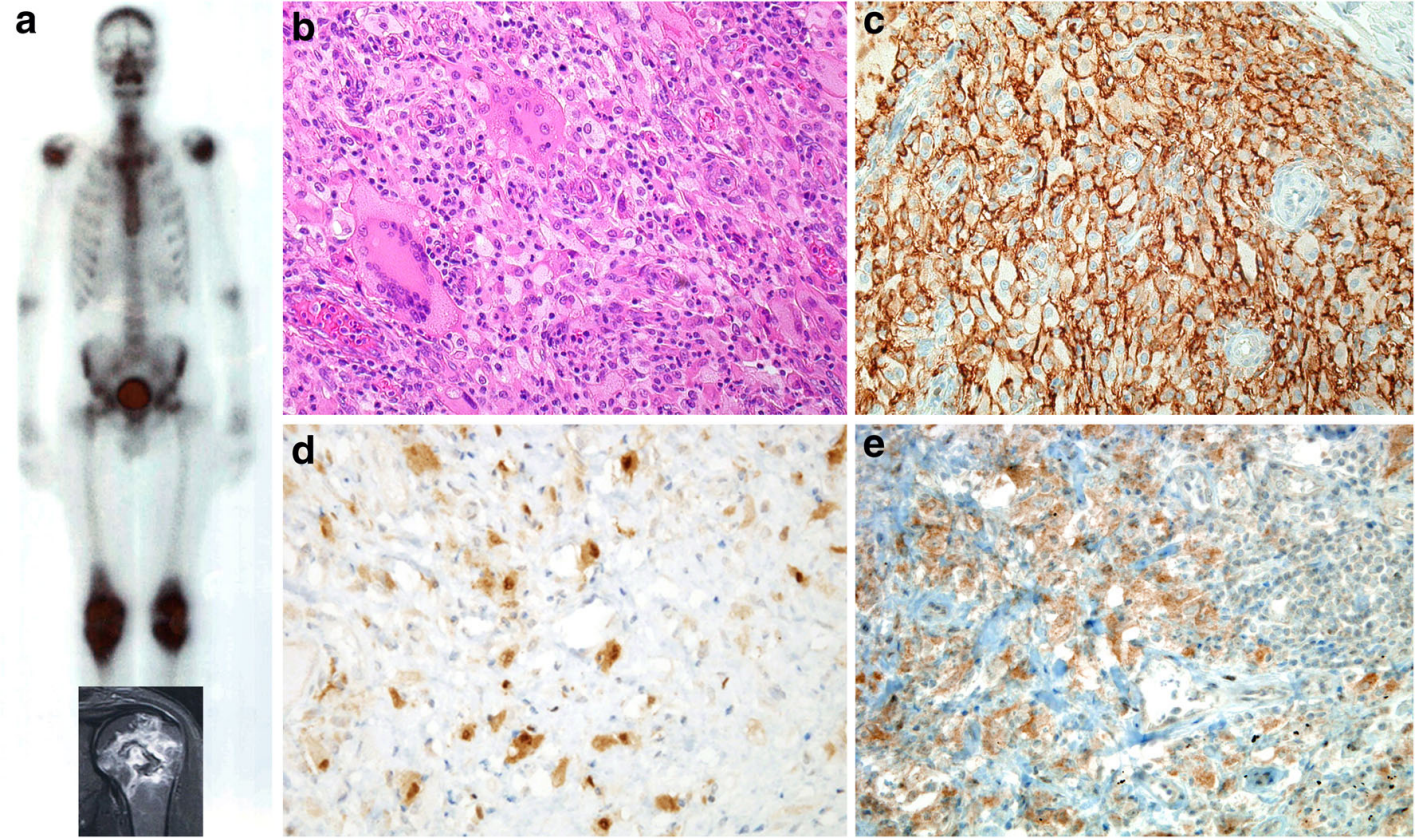
**a** Characteristic imaging appearance of Erdheim-Chester disease (ECD) on whole body scintigraphy and MRI (case 8). **b** Conventional H&E histopathology of an ECD lesion (× 360, case 12). **c** Membranous positivity for CD14 (× 400, case 7). **d** Occasional partial positivity for S100 (× 360, case 13). **e** Characteristic granular positivity for BRAF^V600E^ in a mutant case (× 360, case 13)

*ECD* is the most intriguing histiocytic disorder affecting the BM from the panel’s point of view. *ECD* is a histiocytic neoplasm involving the skeleton, particularly the long bones, with a distinctive radiological presentation of symmetrical diaphyseal and metaphyseal cortical bone sclerosis (Fig. 2a), the cardiovascular system, the lungs, the retroperitoneum with so-called coated aorta and hairy kidney, the CNS, and the skin. It is composed of foamy histiocytes (FXIIIa+/CD14+/CD68+/CD163+/langerin−) and Touton giant cells. In over 70% of cases, targetable MAPK pathway or PI3K-AKT pathway gene mutations can be detected [11, 12, 41]. As in LCH, the multisystem involvement by ECD and evidence from 4/7 cases submitted to this workshop, which showed concurrent or metachronous occurrence of myeloid neoplasms [AML or chronic myelomonocytic leukemia (CMML)], suggest that, like multisystemic LCH, ECD may also be a neoplastic hematopoietic disorder derived from aberrant progenitor cells which in this case have acquired MAPK pathway or PI3K-AKT pathway gene mutations.

The next section summarizes the most important features of the submitted workshop cases and focuses on a few important practical lessons learned, based on these cases.

## Cases and discussion

Among a total of 68 workshop cases of histiocytic proliferations in the BM, 17 were considered to be histiocytic neoplasms. Table 1 summarizes these cases.

Two of the 9 workshop submissions with ECD proved to be the same patient (case 12) that had been treated in different hospitals. All eight patients with ECD presented with multiple bone lesions and varying involvement of soft tissues, mainly retroperitoneum and mesentery, lungs, and/or orbital fat (case 7). Clinical symptoms consisted of localized or generalized bone pain, dyspnea, proptosis, or B symptoms. The BM showed accumulations of bland histiocytes, including cells with foamy cytoplasm, in an often-fibrotic background (Fig. 2b, case 12). The histiocytes expressed CD14, CD68, CD163, FXIIIa and fascin (Fig. 2c). Protein expression of BRAF^V600E^ was shown in 2 mutant cases (cases 9 and 13). Two cases were focally S100 positive (Fig. 2d, e; cases 10 and 13), and one of the S100-positive cases displayed some degree of hemophagocytosis (case 10); CD1a was always negative. When performed, cytogenetics was unremarkable, while the *BRAF*^V600E^ mutation was found in 4 of 7 tested cases (57%), and 1 case (case 12) displayed the pathogenic *NRAS*^Q61R^ mutation [42].

**Fig. 3:**
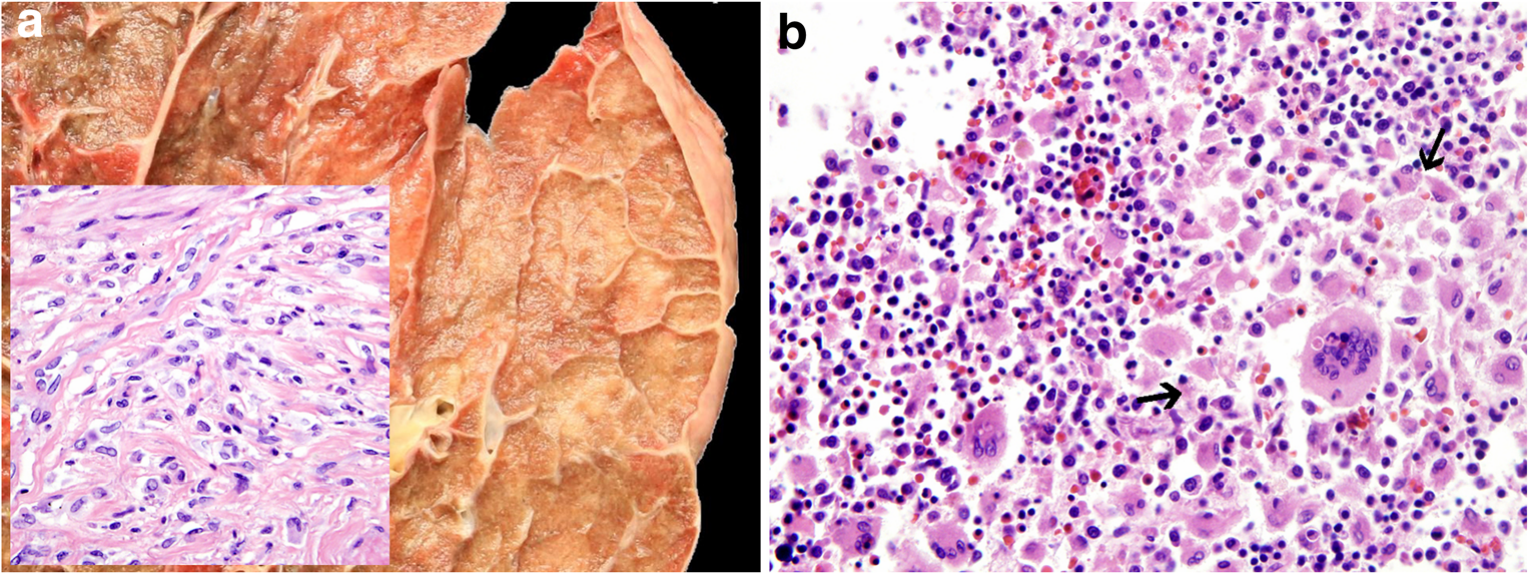
**a** Case 10. Diffuse pulmonary infiltration by a xanthomatous proliferation composed of Erdheim-Chester disease-like histiocytes (insert). **b** Bone marrow infiltration by an undefined myeloid neoplasm with histiocytic appearance (area between arrows) and visible hemophagocytosis, × 360

Remarkably, 4/7 ECD patients suffered from synchronous or metachronous myeloid neoplasms, CMML (*n* = 2, cases 11 and 12) and AML with at least phenotypic monocytic differentiation (*n* = 2, cases 13 and 14), and one patient suffered from an undefined myeloid neoplasm with accompanying HLH, which was considered by the submitter to represent an ECD (*n* = 1, case 10). These patients were all male and more than a decade older (mean age 65.4 years), than those suffering from ECD alone with a male:female ratio of 2:1 and a mean age of 51.3 years. All four cases had a *BRAF*^V600E^ or *NRAS*^Q61R^ mutation and showed the same mutation in the ECD lesions and in the myeloid neoplasm. These were twice combined with additional mutations, including *TET2* and *SRSF2* mutations in a CMML case, and *ASXL1*, *TET2*, and *U2AF1* mutations in the disseminated case of an undefined myeloid neoplasm accompanied by HLH (Fig. 3a, b; case 10). The exact classification of the latter was difficult because of the rapid death of the patient due to disseminated intravascular coagulation and HLH. In addition, the histiocytes of this case were S100 positive and displayed hemophagocytic activity, so that, despite suggesting ECD in many aspects, the case was considered by the panel to be better classified as HLH associated with undefined myeloid neoplasm.

Treatment of the ECD cases included mainly steroids, interferon-α, methotrexate, and chemotherapy. The treatment and the prognosis in composite cases were mainly determined by the accompanying myeloid neoplasm. Noticeably, the case bearing the highest number of mutations, particularly of mutations known to be associated with poor prognosis in myeloid disorders (e.g., *ASXL1* and *U2AF1*) [43], had a very aggressive clinical behavior.

**Fig. 4:**
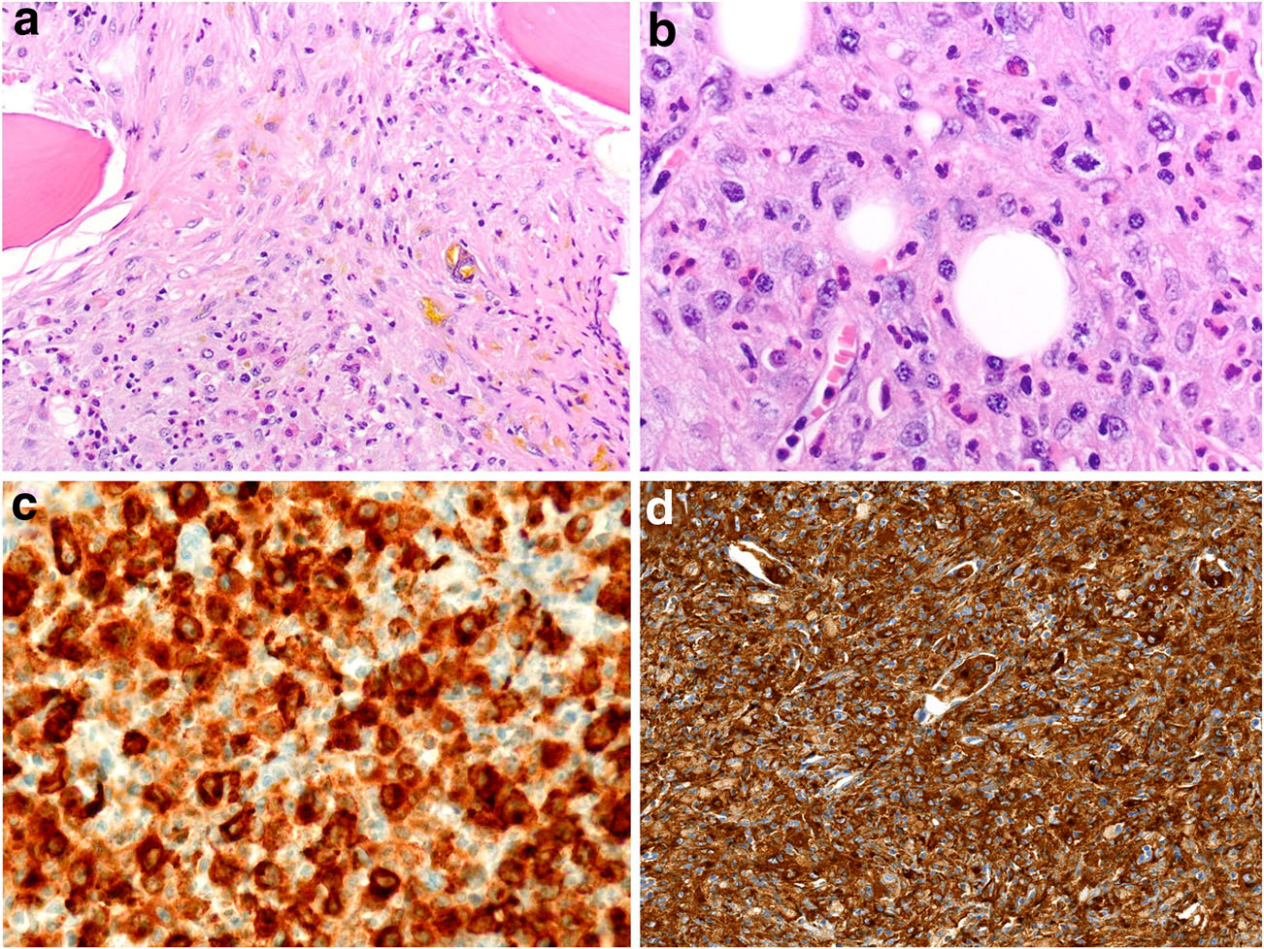
**a** Case 3. Histiocytic sarcoma involving the bone marrow, × 100. **b** Detailed view of atypical large cells with prominent nucleoli and admixed eosinophils, × 360. **c** Positivity for CD68, × 360. **d** Positivity for CD163, × 200

Three cases considered to represent “true” HS were submitted to the workshop (cases 1, 2, and 3). Shared clinical features of these cases included involvement of the liver and/or the spleen and systemic symptoms such as fever, fatigue, weight loss, and/or hemophagocytosis. The BM and other involved tissues showed aggregates of large bizarre histiocytes with abundant pale cytoplasm and pleomorphic nuclei with occasionally pronounced nucleoli and numerous mitoses (Fig. 4a, b). The tumor cells expressed CD68 and CD163 (Fig. 4c, d), and lysozyme and fascin, when tested. One of these cases (case 1) illustrated the sometimes challenging differentiation between HS and other myeloid malignancies with monocytic/histiocytic differentiation. The tumor cells in this case were rather small (< 20 μm) and monotonous and expressed CD33, thereby not fulfilling the morphologic and phenotypic requirements for HS [12, 18, 44–46]. The case was therefore reclassified by the panel as AML with monocytic differentiation. One of the other HS cases had clinical records and showed an aggressive clinical course, dying before diagnosis (case 3). No genetic aberrations were found in the HS cases by karyotyping (2/3 cases) or sequencing (21 gene myeloid panel in 1 case). Yet, based on a few studies with mutational data on HS published to date, the Ras/Raf/MEK/ERK signaling pathway in HS seems also to be recurrently targeted by mutations: the *BRAF*^V600E^ mutation was detected in 5 of 8 HS [47]; a case of *BRAF*^F595L^ mutation along with mutant *HRAS* [48] has been documented; *BRAF*^G466R/G464V/N581S^ mutations and *KRAS*, *PTPN11*, *TP53*, *PTEN*, and *PIK3CA* were recently published [49]. Interestingly, these types of *BRAF* mutations have been reported in carcinoma and melanoma, but not in other histiocytic tumors [11]. Very recently, the report of the 2016 EAHP workshop on histiocytic neoplasms involving the lymph nodes documented 7 HS, of which 2 had *BRAF*^V600E-^, one a *TP53* and one a *MAP2K1* mutation (along with *BCL2*, *BCL10*, *CDKN1B*, *CKIT*) [35]. Since the prognosis of HS is dismal with current treatment strategies and several of the above-mentioned mutations are actionable, their detection and specific targeting yield treatment potential, as already suggested by single-case observations [35, 39, 40].

**Fig. 5:**
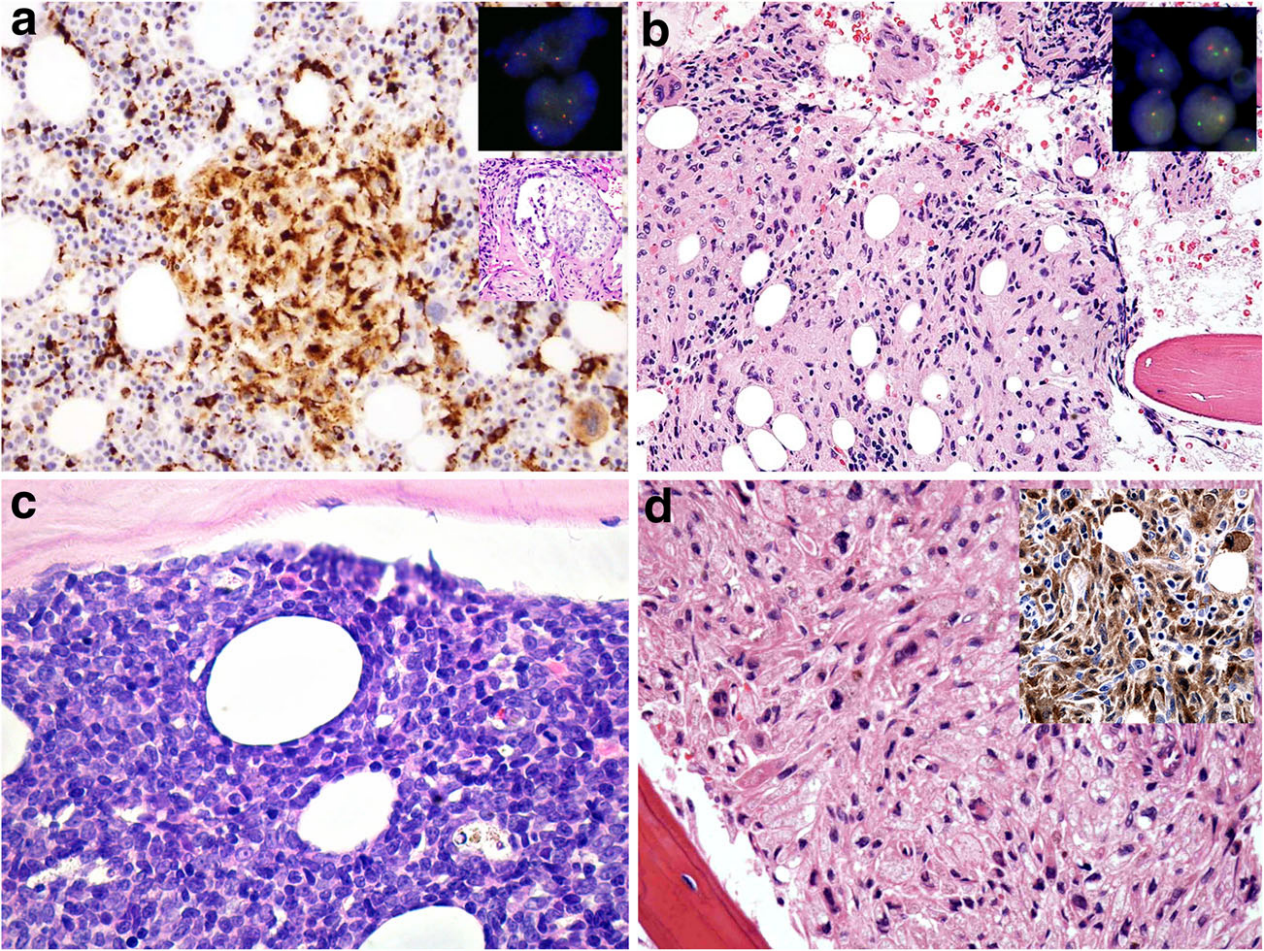
**a** Case 4. CD68-positive cells of a HS involving the bone marrow in a patient suffering from a mediastinal germ cell tumor, displaying three *ETV6* signals on an interphase FISH suggestive of i(12p) (insert), confirming  clonal relationship to the germ cell tumor, × 200. **b** Case 5. HS in the bone marrow of a patient with flow cytometrically proven follicular lymphoma [corresponding to the crushed, small, CD20-positive (not shown), lymphoid cells seen in the upper right part of the microphotograph near the insert], displaying rearranged *BCL2* signals on an interphase FISH (insert), indicative for a common clonal origin of both neoplasms, × 200. **c** Case 6. Bone marrow involvement by acute T lymphoblastic leukemia (T-ALL), × 400. **d** Atypical histiocytic proliferation with juvenile xanthogranuloma phenotype (insert: FXIIIa) that developed 8 months after remission of the T-ALL, × 360

Three histiocytic neoplasms occurred in the context of other neoplastic diseases. Case 4 represented the rare, yet historically well-documented combination of a HS and a mediastinal germ cell tumor. The patient presented with pain under the left rib arch, bicytopenia, splenomegaly, and a mediastinal tumor, consisting of a mixed germ cell tumor (teratoma and yolk sack tumor). The spleen and the BM of the patient were involved by concomitant HS with prominent hemophagocytic activity (Fig. 5a). Interphase FISH, applying a dual-color break-apart probe for the *ETV6* locus at 12p13.2, showed signals suggestive of i(12p) in both neoplastic components, thus proving their clonal relationship (Fig. 5a insert). Case 5 illustrated the well-known transformation of FL to HS [33, 34]. Differently from previous cases, the patient presented with multiple liver lesions due to the HS, and the FL component was only found in the staging BM biopsy along with the HS (Fig. 5b). FISH analysis demonstrated *BCL2* rearrangements in both components, proving their relationship. The last case in this series (case 6) was an atypical histiocytic proliferation with JXG phenotype involving the BM with subsequent spread to the skin, liver, spleen, and pancreas, after remission of T-ALL (Fig. 5c). The BM showed massive involvement by JXG-like lesions, displacing the hematopoiesis (Fig. 5d). Attempts to study the molecular relationship of the JXG-like component with the previous T-ALL that was clonal for the T cell receptor genes and had a recurrent del(9)(p13) failed since the histiocytic lesion showed no clonal rearrangement and did not display del(9)(p13). A possible role of the initial myelotoxic therapy in the development of the JXG-like lesions, similar to documented LC proliferations [50–52], has to be also taken into consideration in this particular case.

Together with 14 analogous cases submitted to the EAHP workshop on histiocytic neoplasms involving the lymph nodes [35], the above cases add further strong evidence in support of the frequent clonal relationship between mature B cell lymphomas and T-ALL/lymphoblastic lymphomas on the one hand and histiocytic neoplasms on the other [33, 34, 53–60]. While transdifferentiation of a neoplastic lymphoid cell after loss of the lineage transcription program has initially been proposed as an explanation [33, 45, 54], evidence now points towards divergent differentiation of a common progenitor cell into multiple lineages as an alternative pathogenic mechanism [34, 58, 60].

**Fig. 6:**
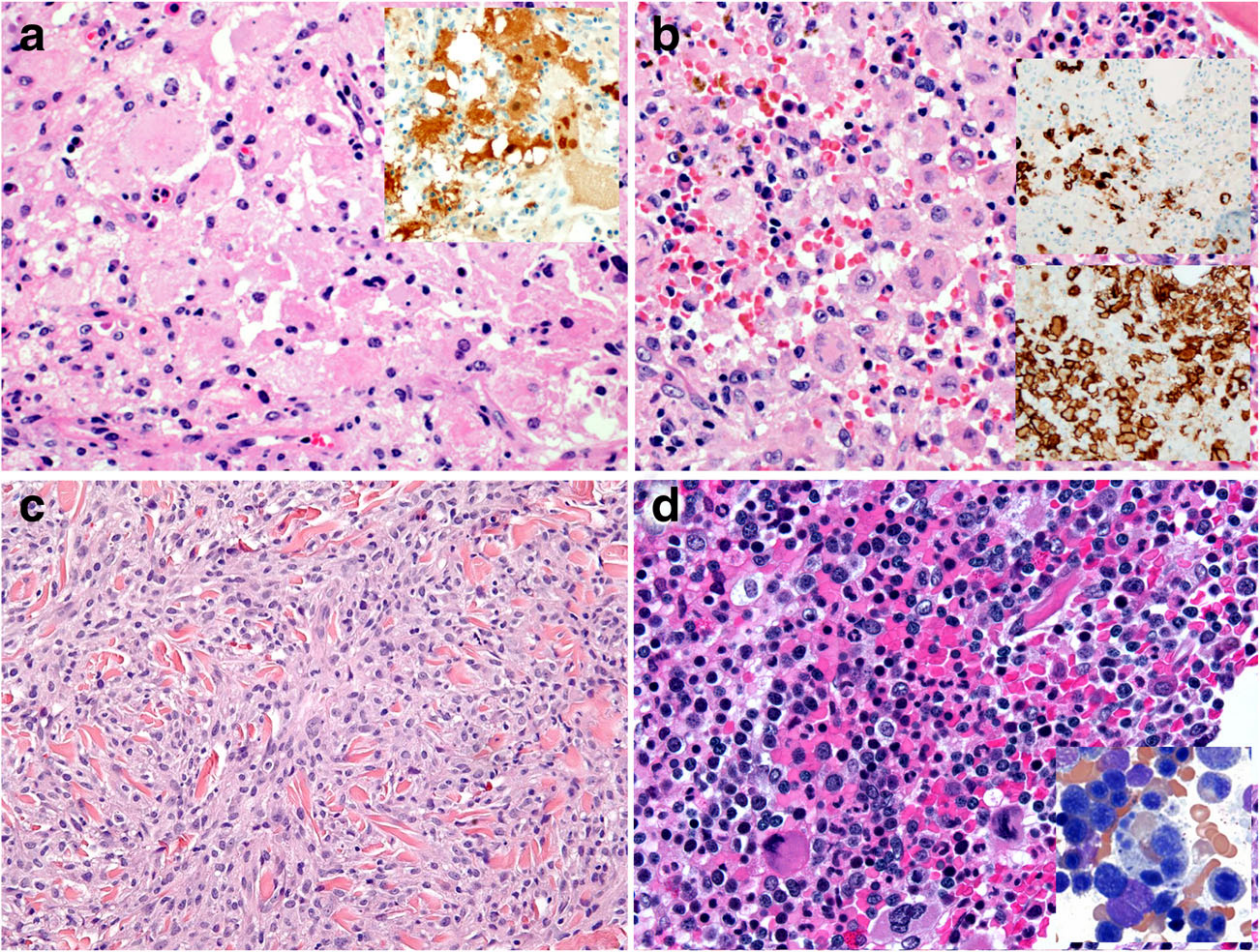
**a** Case 15. Rosai-Dorman disease with aggressive features diffusely spreading to the bone marrow (insert: S100 staining), × 360. **b** Case 16. Langerhans cell sarcoma in the bone marrow expressing langerin (upper insert) and with aberrant expression of CD163 (lower insert), × 360. **c** Case 17. Disseminated juvenile xanthogranuloma, skin lesion (×200), associated with **d** bone marrow changes with reactive erythroid hyperplasia and hemophagocytosis (note “strawberry-like cells” with engulfed erythrocytes), × 360

Two additional cases deserve special attention because of specific diagnostic dilemmas. The first one (case 15) was RDD in a patient with an uncommon generalized presentation involving multiple lymph nodes and the skeleton (Fig. 6a), pointing towards the importance of distinguishing classical RDD cases, with single or regional lymph node involvement and a self-limiting clinical course, from extranodal cases [12]. The second one (case 16) was thought by the submitter to represent a mixed LC/non-LC sarcoma since a subset of the infiltrate expressed LC markers, while other cells expressed CD163. Yet, both components showed a *BRAF*^V600E^ mutation and were intermingled (Fig. 6b). Therefore, the panel felt that the diagnosis was LC sarcoma with aberrant expression of CD163, fitting with the current view of the histogenesis of “composite histiocytoses” from a histiocytic cell pool bearing the *BRAF*^V600E^ or equivalent mutations and retaining some plasticity [28, 61, 62].

Finally, a rather prototypic case (case 17) of disseminated JXG (Fig. 6c, d) with cytogenetic evidence of clonality [63] that was accompanied by HLH and resolved after HLH protocol treatment rounded up the workshop. The authors of this case had comprehensively investigated the important differential diagnoses of juvenile myelo-monocytic leukemia, neurofibromatosis type 1, and familial HLH by targeted massive sequencing approaches.

## Lessons learned

The most remarkable observation of this workshop on neoplastic histiocytic disorders involving the BM, and the *first lesson learned*, was the high concurrence of ECD and myeloid neoplasms, mainly CMML or AML with monocytic/histiocytic phenotype. Five of the 8 workshop cases of ECD and analogous lesions involving the BM (56%) showed evidence of mostly clonally related myeloid neoplasms (4 cases). This new observation was confirmed in an ASH abstract by Durham et al. [64]. Their series of 190 adult histiocytosis patients showed co-existing myeloid malignancies in 9.5%, usually patients with ECD and/or LCH, combined with myelodysplastic-myeloproliferative neoplasms, classical myeloproliferative neoplasms, or myelodysplastic syndromes. Only general data were provided on co-existing histiocytosis-associated mutations (*BRAF*, *MAP2K1*, *NRAS,* or *KRAS)* and myeloid-associated mutations (*ASXL1*, *CALR*, *IDH1/2*, *JAK2*, and *TET2*) in the same patients, without information on the tested material or exact combinations of mutations. In our workshop cases, molecular characterization of both components of these cases provided evidence of the clonal relationship between ECD and the concurrent or evolving myeloid neoplasms, ranging from a single (*BRAF*^V600E^ or *NRAS* ^Q61R^) to multiple shared mutations (*ASXL1*, *BRAF*^V600E^, *TET2*, and *U2AF1* or *BRAF*^V600E^, *SRSF2*, and *TET2*, respectively) in 2 cases each. Since at least some of these mutations are actionable and patients qualify for tailored therapies [28], patients have to be tested with gene panels whenever possible when treatment is planned, remembering that mutation pattern can change during such targeted therapies [41].

An immediate practical aspect and the *second lesson learned*, yet admittedly based on this limited workshop series: ECD patients with associated myeloid neoplasms were exclusively male and more than a decade older than those affected by ECD alone, while the latter showed the age and sex distribution reported by the WHO [18]. Based on this observation, we suggest excluding co-existent myeloid neoplasms at the initial staging of elderly male patients suffering from ECD. As expected in composite cases, the treatment and the prognosis were mainly determined by the accompanying myeloid neoplasm.

Observations on the few submitted HS cases reconfirmed an *old lesson learned* for this entity: its status as a highly aggressive neoplasm often accompanied by organomegaly and hemophagocytosis [12]. Meticulous phenotypic studies are required to exclude other diseases such as anaplastic lymphomas, sarcomas, melanomas, and AML with monocytic/histiocytic differentiation, which can all mimic HS histopathologically. Among the markers required to be negative, CD33 deserves special attention as a marker precluding the diagnosis of HS [41, 58, 59], despite not being listed by the WHO in the current classification [18].

A *lesson to be learned* from the histiocytic proliferations combined with FL, germ cell tumors, or T-ALL is to look systematically for underlying lymphomas in any cases of HS. B or T cell gene rearrangement studies and FISH can prove clonal relationship in such instances.

In summary, a spectrum of neoplastic histiocytic disorders can affect the BM, mainly as multisystem disorders. A substantial proportion is combined with—mostly—clonally related lymphoid or myeloid diseases (particularly ECD). Applying the WHO criteria and integrating clinical, laboratory, and radiologic findings, classification of the respective entities is usually possible. Yet in HS—lacking disease-defining clinical, laboratory, radiologic, and molecular features—broad phenotypic panels must be applied. Deregulation of the Ras/Raf/MEK/ERK signaling pathway and, to a lesser extent, of the PI3K pathway by recurrent mutations is of pathogenic and diagnostic importance in histiocytic neoplasms, as illustrated by many cases presented at the workshop. Therefore, extensive mutational screening with properly designed gene panels is highly recommended, not only for diagnostic but also for therapeutic purposes.
